# Symptomatic aggravation after corticosteroid pulse therapy in definite sporadic Creutzfeldt-Jakob disease with the feature of Hashimoto’s encephalopathy

**DOI:** 10.1186/s12883-014-0179-y

**Published:** 2014-09-08

**Authors:** Jae-Won Jang, So Young Park, Young Ho Park, Jung E Kim, SangYun Kim

**Affiliations:** Department of Neurology, Kangwon National University Hospital, Chuncheon, Republic of Korea; Clinical Neuroscience Center, Seoul National University Bundang Hospital, 300 Gumi-dong, Bundang-gu, Seongnam-si, Gyeonggi-do 463-707 Republic of Korea; Department of Neurology, Seoul National University College of Medicine, Seoul, Republic of Korea

**Keywords:** Creutzfeldt-Jakob disease, Hashimoto’s encephalopathy, Corticosteroid, Seizure

## Abstract

**Background:**

Creutzfeldt-Jakob disease and Hashimoto’s encephalopathy often show similar clinical presentation. Among Creutzfeldt-Jakob disease mimics, Hashimoto’s encephalopathy is particularly important as it is treatable with corticosteroids. Thus, in cases of middle-aged woman diagnosed with probable Creutzfeldt-Jakob disease and who exhibit high titers of antithyroid antibodies, corticosteroid pulse therapy is typically performed with expectations of near complete recovery from Hashimoto’s encephalopathy. Herein, we provide the first case report that exhibited a negative effect of corticosteroid pulse therapy for a patient with Creutzfeldt-Jakob disease with features of Hashimoto’s encephalopathy.

**Case presentation:**

We report a case of 59-year-old Asian woman with blurred vision, dysarthria, myoclonus, and rapidly progressive dementia. Cerebrospinal fluid showed 14-3-3 protein positive. Electroencephalogram showed periodic sharp waves (1.5 Hz) at the bilateral frontal or occipital areas. Magnetic resonance imaging showed high signal intensities at the bilateral cerebral cortex, caudate nucleus, and putamen. The patient was diagnosed with probable Creutzfeldt-Jakob disease. However, serum analysis showed a high titer of antithyroid antibodies. We started corticosteroid pulse therapy with subsequent aggravation of seizure activity including generalized myoclonus, epilepsia parialis continua, and ballistic dyskinesia, which was effectively treated with clonazepam.

**Conclusion:**

We provide evidence of a case of Creutzfeldt-Jakob disease that exhibited clinical deterioration after corticosteroid therapy. Although histopathological confirmation with brain biopsy is not easily available in Creutzfeldt-Jakob disease patients, selective initiation of corticosteroid pulse therapy should be considered in cases of uncertain diagnosis for differentiation with Hashimoto’s encephalopathy.

## Background

Creutzfeldt-Jakob disease (CJD) is fatal prion disease characterized by rapidly progressive dementia, myoclonus, pyramidal and extrapyramidal signs, visual field defect, and cerebellar symptoms [1]. Hashimoto’s encephalopathy is corticosteroid-responsive autoimmune encephalitis with antithyroid antibodies that may have overlapping clinical symptoms with CJD, especially in the early phase of disease [2]. Hashimoto’s encephalopathy is more common in female individuals, typically presenting in middle age with a fluctuating encephalopathy including rapid progressing dementia, seizure, psychiatric manifestations, myoclonus, ataxia, stroke-like episodes, and coma, although the clinical phenotype is variable [3]. Cerebrospinal fluid (CSF), electroencephalogram (EEG), and brain magnetic resonance imaging (MRI) findings are non-specific, and CJD diagnosis is based on an appropriate clinical phenotype with exclusion of other conditions, in addition to raised anti-thyroid antibodies (Tg-Ab and TPO-Ab). However, anti-thyroid antibodies are not specific to Hashimoto’s encephalopathy and are common in healthy elderly people [4]. There is no obvious correlation between antibody levels and disease severity, and it is likely that the antibodies are not pathogenic but simply an epiphenomenon reflecting an underlying autoimmune inflammatory state [5]. Thus, it is widely considered that Hashimoto’s encephalopathy should be relabeled as ‘corticosteroid-responsive encephalopathy associated with autoimmune thyroiditis’ [6], and that lack of such corticosteroid response should prompt review of diagnosis [4]. Among CJD mimics, Hashimoto’s encephalopathy is particularly important as it is treatable with corticosteroids [7]. In our thorough review of the literature on CJD mimicking Hashimoto’s encephalopathy or Hashimoto’s encephalopathy mimicking CJD, we found no evidence of definite markers for differential diagnosis of CJD and Hashimoto’s encephalopathy [2,8–14]. Here, we describe a case of a patient with definite CJD with high titers of anti-thyroid antibodies whose clinical symptom was aggravated as a dominant seizure after initiation of corticosteroid pulse therapy.

## Case presentation

A 59-year-old woman was admitted because of blurred vision, dysarthria, myoclonic movement of bilateral upper and lower limbs, and rapidly progressive dementia. The symptoms had started approximately 1 month prior and progression was so rapid that she became akinetic and occasionally showed just a slight smile as a response to questioning over the last 2 weeks.

**Figure 1:**
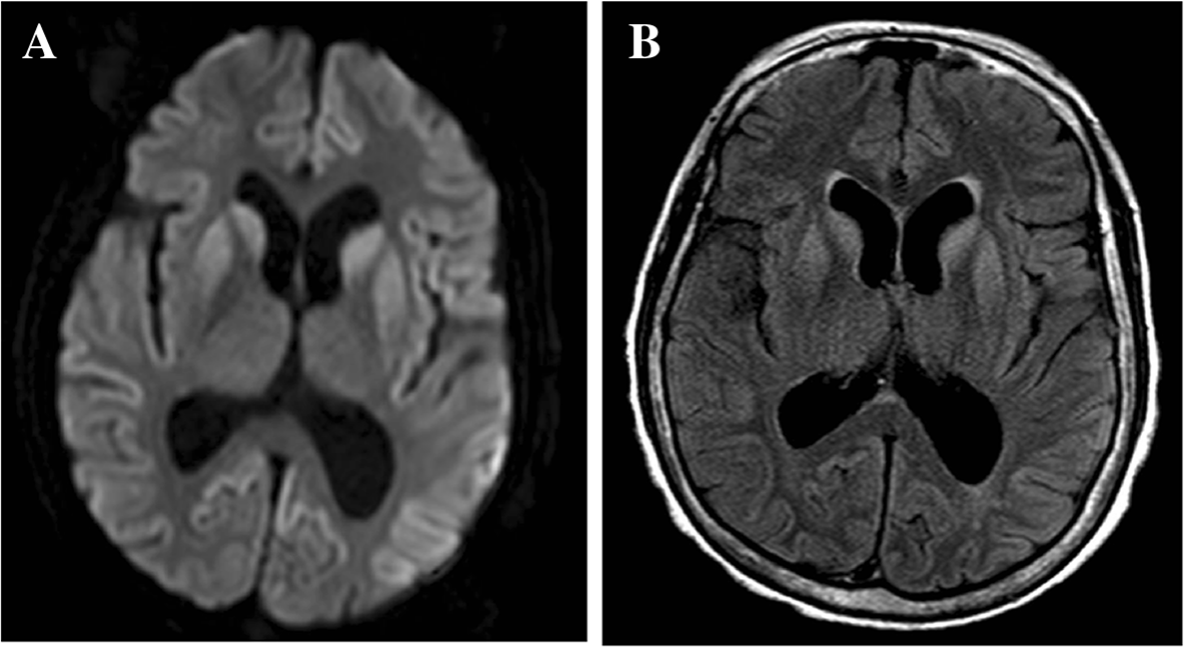
**Patient MRI scans.** Bilateral high signal intensities in the cortex, caudate nucleus, and putamen on DWI **(A)** and FLAIR **(B)**.

Her CSF was acellular with no evidence of bacterial or viral infection, but her 14-3-3 protein was positive. EEG showed periodic sharp waves (1.5 Hz) at the bilateral frontal or occipital areas with predominance in the left side. Contrast-enhanced MRI showed high signal intensities at the bilateral cerebral cortex, caudate nucleus, and putamen with left predominance (Figure 1). She was diagnosed with probable CJD according to the World Health Organization diagnostic criteria for sporadic CJD [15]. Sodium valproate (1200 mg/day) and clonazepam (0.5 mg/day) were used for intermittent myoclonic jerk. Laboratory analysis showed high titers of anti-thyroid peroxidase antibody (TPO-Ab) (1370 IU/mL; normal value <60 IU/mL) and anti-thyroglobulin antibody (Tg-Ab) (167 IU/mL; normal value <60 IU/mL). However, the levels of thyroid-stimulating hormone (2.24 IU/mL; normal value 0.4–4.0 IU/mL) and FT4 (1.23; normal value 0.89–1.79) were normal. Follow-up laboratory study shows persistent high titers of TPO-Ab, while the titer of Tg-Ab was fluctuating around the normal upper limit. Thyroid sonography showed no gross abnormality.

**Figure 2:**
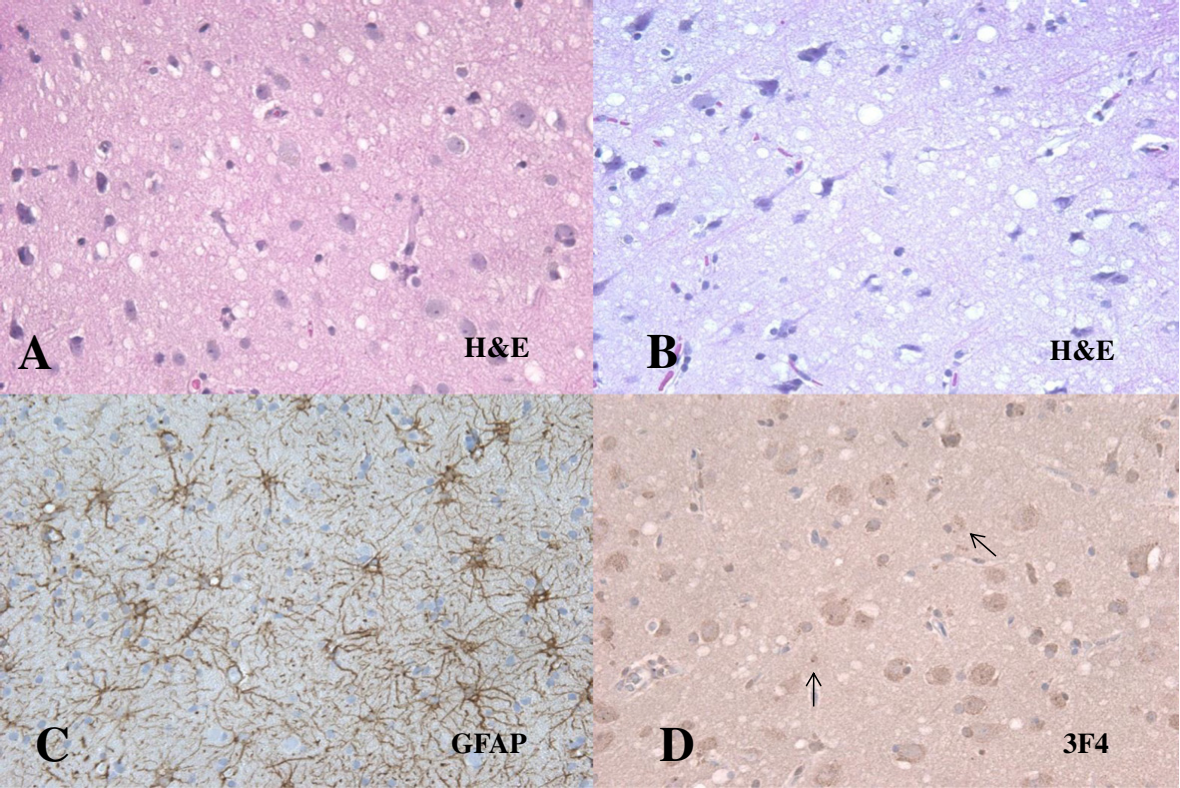
**Brain biopsy finding of the patient.** Spongiform changes with some neuronal cell loss and degeneration in H&E **(A, B)**. Increased gliosis in GFAP **(C)** and positive PrP^sc^ in 3 F4 **(D)**.

Although she was diagnosed as probable CJD, corticosteroid pulse therapy with methylprednisolone (1 g per day) was started on the 6^th^ day of admission for differential diagnosis with Hashimoto’s encephalopathy, which is known to show excellent response to corticosteroid therapy. We observed alertness and myoclonic movement after careful initiation of corticosteroid therapy. Over the 2 days after starting corticosteroid treatment, she was in a akinetic mute state with no response to pain or visual response. We also observed two episodes of generalized clonic movement with eyeball deviation. We elevated the dose of sodium valproate, titrated to 1800 mg/day, without subsequent improvement of myoclonic jerk. Her alertness kept worsening with aggravation of myoclonus, and she had a first generalized tonic-clonic seizure on the 4^th^ day of corticosteroid therapy. We added levetiracetam (1000 mg/day) and elevated the dose of sodium valproate (2400 mg/day). However, the frequency of seizures was increased and the EEG showed more periodic sharp waves in the bilateral hemispheres with background slowing compared with the previous EEG. Generalized myoclonic movement was observed almost continuously when she was awake. Thus, we elevated the dose and frequency of clonazepam, which effectively alleviated the myoclonic jerk, although she subsequently exhibited decreased mentality. As the five days of corticosteroid pulse therapy were suspected to aggravate the seizures, we performed a brain biopsy with family consent to confirm a diagnosis of CJD. Brain biopsy revealed neuronal loss, vacuolation and gliosis (Figure 2). Western blot demonstrated a protease-resistant pathogenic form of prion protein in the brain tissue. Genetic analysis demonstrated no known mutation in the PRNP gene. A diagnosis of definite CJD was made based on the above findings.

## Discussion

CJD and Hashimoto’s encephalopathy often share similar clinical features. However, there are no definite markers for differential diagnosis except for brain biopsy (Table 1). Clinical manifestation, EEG, brain imaging, and CSF findings including 14-3-3 protein can overlap between both disorders. The response to corticosteroid therapy is generally considered the only effective method for differential diagnosis before brain biopsy. Thus, in middle-aged female patients with a high titer of anti-thyroid antibodies, and who show dementia, myoclonus, ataxia, and altered consciousness, there is a mandatory recommendation to start corticosteroid therapy to rule out Hashimoto’s encephalopathy [2]. Thus, we performed corticosteroid pulse therapy in our case.

**Table 1:** **Previously reported cases with the feature of CJD and HE in need of differential diagnosis**

**Year/author**	**Final diagnosis**	**Clinical findings**	**Corticosteroid response**	**EEG finding**	**Brain image**	**CJD work-up**		**Hashimoto encephalopathy work-up**	
						**CSF study**	**Biopsy/gene**	**Lab**	**US/SCAN/FANA**
2004/Cho *et al.* [14].	CJD	66/F	−	Bilateral frontal SWC (0.5–2 Hz)	MRI (DWI): HSI at cortex, caudate nucleus, putamen	14-3-3: +		TG-AB: WNL	US: Chronic thyroiditis
		Dementia, parkinsonism, visual symptom, ataxia, myoclonus, akinetic mutism			SPECT/PET: Rt. Hemisphere: ↓			TPO-AB: ↑	FNA: PMN, lymphocyte
2003/Cossu *et al.* [2].	CJD	61/F	−	Periodic triphasic wave	MRI: normal	14-3-3: +	Biopsy: typical CJD pattern, PRNP + codon 210, 129	TG-AB: ↑	
		Visual symptom, ataxia, myoclonus, mental change						TPO-AB: ↑	
								TSH : ↑	
								T3, T4 : WNL	
2012/Kondziella et al. [10].	CJD	67/F	−					Anti-thyroid antibody (+)	
	HE	63/F	+						
		Dementia, ataxia, myoclonus							
2008/Cerqueira *et al.* [8].	HE	68/F	+	Occasional sharp waves (2–3 Hz)	MRI (T2WI): HSI at corona radiata, centrum semiovale			T3, T4: WNL	
		Cognitive decline, insomnia, poor appetite, visual hallucination, tremor, gait disturbance, decreased mentality, myoclonus						TSH: ↑	
								TPO-AB: ↑	
								TG-AB: WNL	
2002/Doherty *et al.* [9].	HE	57/F	+	Bihemispheric slowing, triphasic wave	MRI (T2WI): HIS at Lt. medial frontal region	14-3-3: −	Biopsy: spongiform change	TPO-AB: ↑	US: hypoechoic area
		Generalized seizure, hallucination							
2004/Vander *et al.* [13].	HE	58/M	+	Slow background, generalized delta activity	MRI: normal	14-3-3: +		TSH: ↑	
		Confusion, myoclonus, bilateral hyperreflexia, babinski (+) generalized seizure						TG-AB: ↑	
								TPO-AB: ↑	
2011/Santoro *et al.* [12].	HE	66/M	+	Slow theta and delta waves	MRI (DWI, T2WI): HSI at left white matter and bilateral cortical region	14-3-3: −−		TG-AB: ↑	
		Confusion, GTC, fluctuating alertness, myoclonic jerks							
2004/Sakuria *et al.* [11].	HE	79/F	+	Diffuse slowing, periodic synchronous discharge	MRI (T2WI): HSI at periventricular and basal ganglia lesion	14-3-3: +		TG-AB: ↑	
								TPO-AB: ↑	
		Dementia, gait, inactivity, myoclonus						TSH-receptor AB: ↑	
								TPO-AB: ↑	
								TSH-receptor AB: ↑	

*CJD* Creutzfeldt-Jakob disease; *HE* Hashimoto’s encephalopathy; *GTC* generalized tonic-clonic seizure; *DWI* diffusion-weighted images; *HSI* high signal intensity; *Tg-Ab* anti-thyroglobulin antibody; *TPO-Ab* anti-thyroid peroxidase antibody; *US* ultrasonography; *FNA* fine needle aspiration; *PMN* polymorphonuclear leukocytes.

What was unique in our patient is that her myoclonic seizure was distinctly aggravated as epilepsia partialis continua and secondary generalized tonic clonic seizure just after initiation of coritcosteroid pulse therapy. The semiology of her seizure was composed of mostly multifocal myoclonic movement, epilepsia partialis continua, and ballistic dyskinesia, which showed obvious improvement by clonazepam in addition to other anti-epileptic drugs. The response to clonazepam was so marked that an elevation of only 0.25 mg decreased her mentality with subsequent improvement of myoclonic jerk.

To our knowledge, an aggravated seizure just after initiation of corticosteroid pulse therapy in definite CJD has not been previously reported. Although our case was in a course of deterioration, there was clear temporal correlation with the initiation of corticosteroid pulse and abrupt increase in seizure activity. From both acute and chronic models of epilepsy, there is evidence that high corticosteroid levels may exacerbate seizure occurrence [16,17]. Nevertheless, the symptomatology of our patient cannot be fully explained.

Focal motor or generalized seizures have been reported in 15–21% of patients with CJD during the later stage of the disease [18]. However, seizures as the presenting symptom of CJD are uncommon and occur in only approximately 3% of cases [19]. Based on five case reports in the literature, epilepsia partialis continua is reported as a presenting feature of CJD, although none of those cases were related to corticosteroid pulse therapy. The irritative, rather than destructive, nature of the cerebral damage may be the cause of the continuous jerks [20], while the loss of basal ganglia influence on the brain stem can also cause muscular twitches [21]. Our patient also showed ballistic movement, which was reduced by clonazepam treatment. Coexistence of generalized chorea and epilepsia partialis continua as the initial signs was previously reported in probable CJD, although the movement disorder typically appears during the later disease stage [22].

## Conclusion

This is the first definite case of CJD with symptomatic aggravation after corticosteroid pulse therapy for differential diagnosis with Hashimoto’s encephalopathy. In our patient, the high titer of anti-thyroid antibodies may have been an incidental finding, as the thyroid sonography was normal and brain biopsy confirmed final diagnosis as sporadic CJD that was abruptly aggravated by corticosteroids. When a middle-aged woman diagnosed with probable CJD shows a high titer of anti-thyroid antibodies, corticosteroid treatment is typically used as histopathological confirmation with brain biopsy is not easily available, with expectations of near complete recovery in cases of Hashimoto’s encephalopathy. However, if corticosteroid pulse therapy can cause rapid deterioration or seizure aggravation of CJD, the use of corticosteroids in probable CJD patients with high titers of anti-thyroid antibodies should be cautioned. Our patient showed very typical clinical symptoms, and EEG, MRI and CSF findings (14-3-3 protein) of CJD. Thus, it may be reasonable to selectively start corticosteroid pulse therapy in cases of uncertain diagnosis. If seizures are aggravated by corticosteroid treatment, clonazepam may be a good choice to reduce myoclonic jerk, in addition to anti-epileptic drugs. Further studies are required to prove a correlation between corticosteroid treatment and symptomatic aggravation of CJD.

## Consent

Written informed consent was obtained from the caregiver of our patient for publication of this Case report and any accompanying images. A copy of the written consent is available for review by the Editor of this journal.
